# Detection of Human Papillomavirus in Chronic Cervicitis, Cervical Adenocarcinoma, Intraepithelial Neoplasia and Squamus Cell Carcinoma

**DOI:** 10.5812/jjm.9930

**Published:** 2014-05-01

**Authors:** Elahe Mirzaie-Kashani, Majid Bouzari, Ardeshir Talebi, Farahnaz Arbabzadeh-Zavareh

**Affiliations:** 1 Department of Biology, Faculty of Science, University of Isfahan, Isfahan, IR Iran; 2 Pathology Laboratory, Faculty of Medicine, Al-Zahra University Hospital, Isfahan University of Medical Sciences, Isfahan, IR Iran; 3 Department of Operative Dentistry, Faculty of Dentistry, Isfahan University of Medical Sciences, Isfahan, IR Iran

**Keywords:** Polymerase Chain Reaction, Uterine Cervical Neoplasms, Uterine Cervicitis

## Abstract

**Background:**

Cervical cancer is the second most common cancer in women worldwide. Recent studies show that human papillomavirus (HPV) DNA is present in all cervical carcinomas and in some cervicitis cases, with some geographical variation in viral subtypes. Therefore determination of the presence of HPV in the general population of each region can help reveal the role of these viruses in tumors.

**Objectives:**

This study aimed to estimate the frequency of infection with HPV in cervicitis, cervical adenocarcinoma, intraepithelial neoplasia and squamus cell carcinoma samples from the Isfahan Province, Iran.

**Patients and Methods:**

One hundred and twenty two formalin fixed paraffin embedded tissue samples of crevicitis cases and different cervix tumors including cervical intraepithelial neoplasia (CIN) (I, II, III), squamus cell carcinoma (SCC) and adenocarcinoma were collected from histopathological files of Al-Zahra Hospital in Isfahan. Data about histopathological changes were collected by reexamination of the hematoxylin and eosin stained sections. DNA was extracted and subjected to Nested PCR using consensus primers, MY09/MY11 and GP5+/GP6+, designed for amplification of a conserved region of the genome coding for L1 protein.

**Results:**

In total 74.5% of the tested samples were positive for HPV. Amongst the tested tumors 8 out of 20 (40%) of CIN (I, II, III), 5 out of 21 (23.8%) of adenocarcinoma cases and 78 out of 79 chronic cervicitis cases were positive for HPV.

**Conclusions:**

The rate of different carcinomas and also the rate of HPV infection in each case were lower than other reports from different countries. This could be correlated with the social behavior of women in the area, where they mostly have only one partner throughout their life, and also the rate of smoking behavior of women in the studied population. On the other hand the rate of HPV infection in chronic cervicitis cases was much higher than cases reported by previous studies. This necessitates more attention to the role of human papillomaviruses in the their induction in the studied area.

## 1. Background

Human papillomaviruses (HPV) are members of the *Papillomaviridae* family, which are relatively small and non-enveloped (1, 2). After breast cancer cervical cancer represents the second most common malignancy in women worldwide (1, 3-5). Approximately 493000 new cases of cervical cancer and 27300 deaths are reported each year, of these over 80% of HPV-associated diseases are reported from low income countries, where national cervical cancer screening is not performed (4). 

Cervicitis is most often caused by sexually transmitted pathogens of which the main agents are *Chlamydia trachomatis* and *Neisseria gonorrhoeae*. It may also be caused by systemic diseases such as autoimmune diseases, Stevens-Johnson syndrome, neoplasia, mechanical/chemical trauma and viral infection. Herpes simplex virus (HSV) and *Trichomonas vaginalis* may cause ectocervicitis. HPV infection may also cause visible papillary warts, leukoplakia or condyloma (6). In 1981, Zur Hausen et al. reported the detection of HPV DNA in cervical neoplasia (7, 8).

## 2. Objectives

As cervical cancer still remains as one of the major focuses of researchers, this study aimed to estimate the frequency of infection with HPV in cervix tumor samples and also chronic cervicitis cases from Isfahan’s population.

## 3. Patients and Methods

### 3.1. Tissue Samples

One hundred and twenty two formalin fixed paraffin embedded tissue samples of cervicitis and cervical cancers were collected from histopathological files of Al-Zahra hospital in Isfahan. Data about histopathological changes were collected by reexamination of the hematoxylin and eosin stained sections.

### 3.2. Deparaffinization of Tissue Sections

For deparaffinization, ten slices (four micrometers thick) were collected from each block and were subjected to Xylen treatment (1 mL) at 59˚C for 15 minutes in 1.5 mL Eppendorf tubes and centrifuged at 11300 × g for 10 minutes. The procedure was repeated three times, followed by three rounds of washes with 100% ethanol and centrifugation at 9660 × g for ten minutes. Finally, the samples were aired for 30 minutes.

### 3.3. DNA Extraction

The depariffinized tissue sections were treated with 900 µL of a solution containing 50 µL of 5 M NaCl, 200 µL of 0.5 M EDTA and 650 µL of retrieval solution (1 M Tris, 0.5 M EDTA, 10% sodium dodecyl sulfate (SDS)) and thermomixed at 59˚C and 450 rpm for 15 minutes. Next, 90 µL of 0.5 mg/mL proteinase K was added and thermomixed at 59˚C and 500 rpm for three hours. Equal volume of phenol/chloroform/isoamyl alcohol (25:24:1) (Merck, Germany) was added and after five minutes, the solution was centrifuged at 4290 × g for 10 minutes. The upper phase was collected and transferred to another microtube and 0.1 volume of 3 M sodium acetate was added and vortexed for 1 minute then 2 volume of cold 100% ethanol (Merck, Germany) was added and incubated at -20˚C overnight. The precipitated DNA was centrifuged at 4˚C and 9660 × g. The supernatant was discarded and the DNA precipitate was washed once with 75% ethanol. The pelleted DNA was dissolved in 50 µL of distilled water or TE solution (Tris-HCL buffer (10 mM, pH = 8.0) containing 1 mM EDTA) after complete drying.

### 3.4. PCR Amplification

The MY09/MY11 and GP5+/GP6+ primer sets were used in the Nested-PCR (Table 1). The first outer MY primer set amplifies approximately 450 bp within the HPV L1 structural gene (2, 9, 10) while the internal Gp primers generate an approximately 150 bp long fragment from the HPV L1 region within the sequence amplified by the outer primer pair (My09/My11) (2, 11-13). DNA was amplified by two rounds of PCR. In the first round with 3.5 µL of DNA and in the second round with 1 µL of the PCR product in a 25 µL reaction mixture containing 1 U Smar Taq DNA Polymerase (Cinnagen, Iran), 0.4 µM of each primers, 240 µM of each dNTPs, 20 mM of Tris-HCL, 3 mM MgCl_2_, 50 mM KCl and 20 mM ammonium sulfate. 

Thermal cycling conditions in the first round were as follows: denaturation at 95˚C for five minutes followed by 44 cycles of denaturation at 95˚C for 30 seconds, annealing at 62˚C for 45 seconds and extension at 72˚C for 45 seconds. The amplification program was followed by a final extension step at 72˚C for seven minutes. Thermal cycling conditions in the second round were as follows: denaturation of 95˚C for five minutes followed by 44 cycles of denaturation at 95˚C for 30 seconds, annealing at 60˚C for 25 sec and extension at 72˚C for 45 seconds. 

The amplification program was followed by a final extension step at 72˚C for seven minutes. PCR products (10 µL) of the second round were loaded on a 2% agarose gel (Sigma, Germany) containing ethidium bromide and electrophoresed and then DNA was visualized under UV light. Purity of the extracted DNA was estimated as the ratio between spectrophotometric absorbtion at 260 and 280 nm (OD260/OD280) (14). To verify the presence of carcinoma cells in the samples used for the PCR, different prepared paraffin-embedded biopsies were tested for histological evidence of tumors and along the blocks with carcinomas, blocks with normal tissues were also tested.

**Table 1.** Sequence of Primers Used for the Nested PCR

Primers Name	Sequence of Primer
**(Forward) My09 **	CGTCCMARRGGAWACTGATC
**(Reverse) My11**	GCMCAGGGWCATAAYAATGG
**(Forward) Gp5+**	TTTGTTACTGTGGTAGATACTAC
**(Reverse) Gp6+**	GAAAAATAAACTGTAAATCATATTC

### 3.5. DNA sequencing and Computer Analysis of HPV Sequences

The detected bands of about 150 bp were extracted from the agarose gel using the Fermentas DNA extraction Kit Ko513 (Fermentas, Germany) according to manufacturer's instructions. The extracted DNA was sequenced by the Applied Biosystem 3730 DNA Analyzer (Gene service, UK). A WU-BLAST-2 search of the determined sequences against a nucleotide sequence database (EMBL, European Bioinformatics Institute) was performed.

### 3.6. Statistical Analyses

Fisher's Exact test was used for statistical analyses using GraphPad Instat software version 3.05 (GraphPad, USA).

## 4. Results

### 4.1. Histopathological Findings

**Figure 1.**
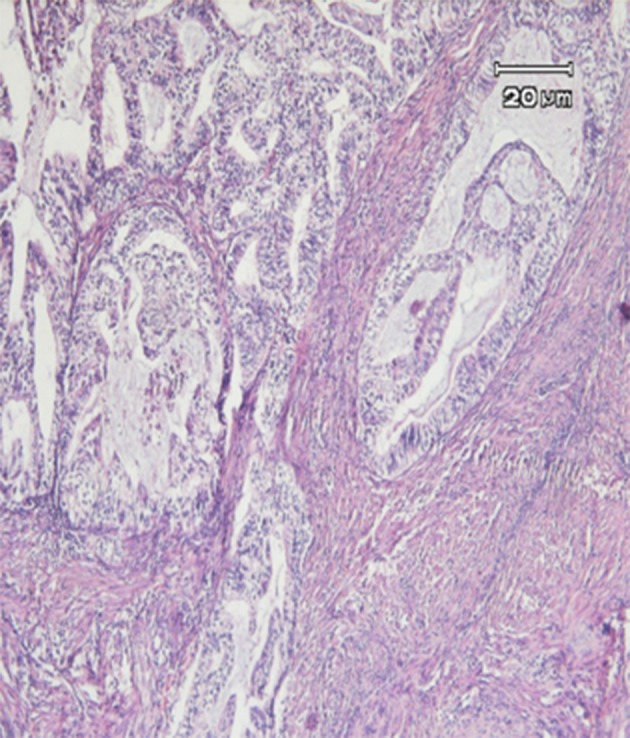
Adenocarcinoma: Malignant Changes Including the Hyperchromatism and Atypism of the Nucleus Are Observed in Cervical Glands

**Figure 2.**
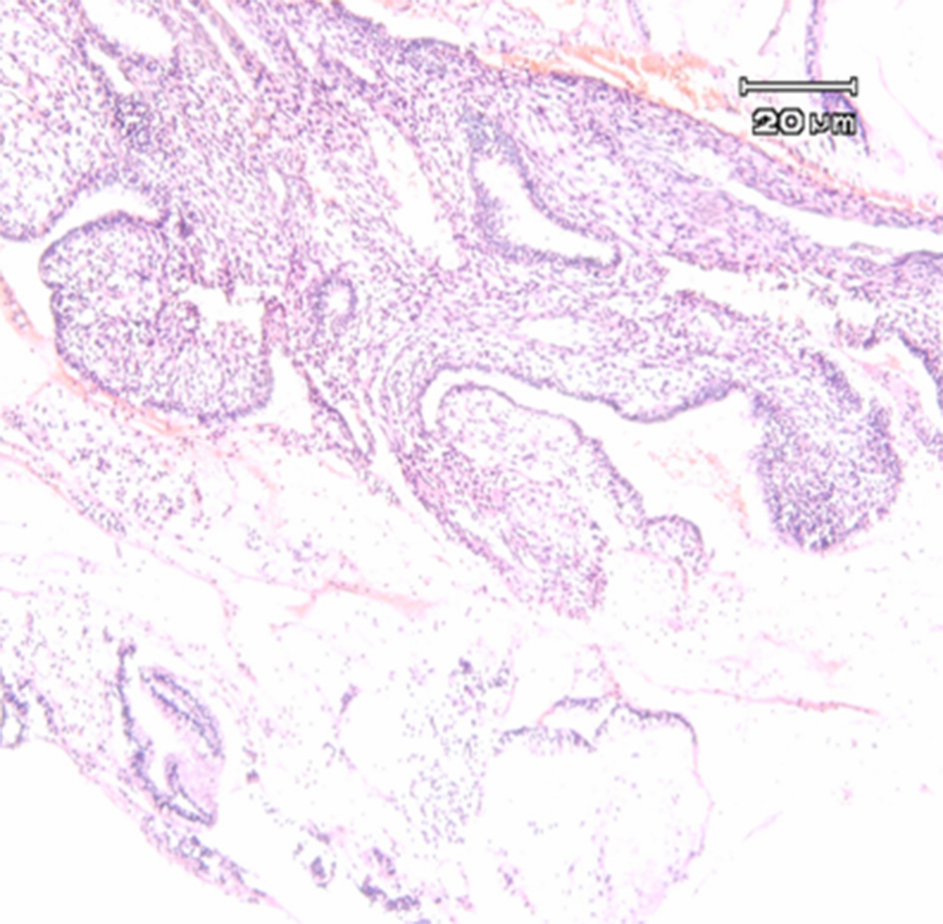
Chronic Cervicitis: Infiltration of Inflammatory Cells (lymphocytes) in Connective Tissue and Metaplasia of Endocervical Epithelium to Squamus Epithelium With no Malignancy Are Observed

Typical histopathological changes were observed in different tumors and cervicitis cases tested (Figures 1 and 2).

### 4.2. PCR

**Figure 3.**
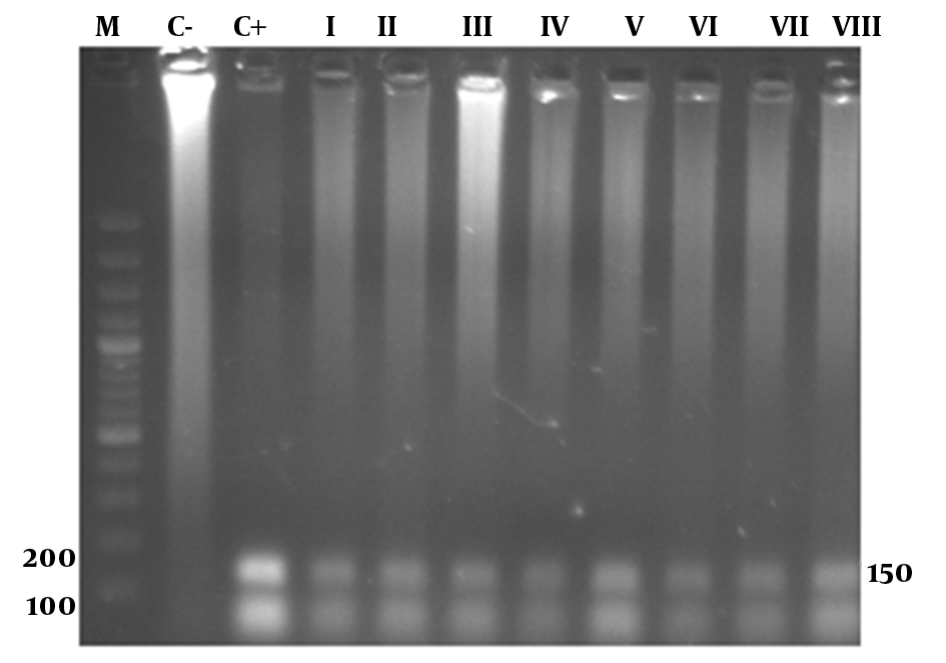
Agarose Gel Showing PCR Products of Targeted DNAs Extracted From Four Tumor Samples and Four Cervicitis Samples M, Marker; C-, Negative control; C+, Positive control; I, II, III, IV, Cervical cancers; V, VI, VII, VIII, Cervicitis.

In the PCR reaction 139 and 157 bp products were observed by gel electrophoresis of positive samples tested (Figure 3).

### 4.3. Alignment

**Figure 4.**
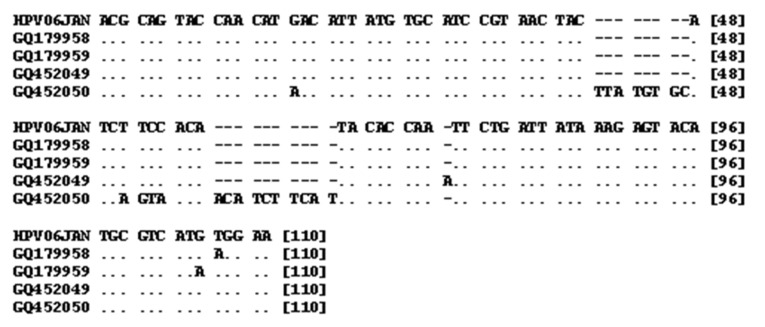
Sequence Alignment of the Four Detected Sequences (Accession Numbers GQ179958, GQ179959, GQ452049, GQ452050) Against Prototype HPV (Accession Number EU911303) (EMBL, European Bioinformatics Institute, WU-BLAST-2)

The PCR sequences obtained included three 139 bp and one 157 bp length sequences with accession numbers (GQ179958, GQ179959, GQ452050, GQ452049) (Table 2). Alignment of 139 bp sequences showed high levels of homology with other isolates of HPV (96-97%). For example, the homologies with isolates VI: EU911303 and VI: S73503 were 97% and 96%, respectively. Alignment of the 157 bp sequence showed a high level of homology with other isolates of HPV (91-93%). For example, the homologies with isolates VI: DQ312263 and VI: S73503 were 91% and 93%, respectively. Sequence alignment of 157 and 139 bp PCR products against prototype HPV (Human papillomavirus isolate 06JAN, accession number EU911303) is shown in Figure 4. As shown by Table 3 the differences among the sequenced PCR products range from 1 to 5%. HPV DNA was detected in 13 out of 43 (32.5%) neoplastic cervical tissue samples and in 78 out of 79 (98.7%) chronic cervicitis cases. The frequency of HPV was significantly higher in chronic cervicitis cases (P < 0.001).

**Table 2.** Sequences of PCR Products (Accession Numbers GQ179958, GQ179959, GQ452049, GQ45205)

Accession No.	Sequence
**GQ452049**	
	1 TTTGTTACTG TGGTAGATAC TACACGCAGT ACCAACATAA CATTATGTGC ATCCGTAACT
	61 ACTTATGTGC ATCAGTAACA ACATCTTCAT TACACCAATT CTGATTATAA AGAGTACATG
	121 CGTCATGTGG AAGAATATGA TTTAAAGTTT ATTTTTC
**GQ452050**	
	1 TTTGTTACTG TGGTAGATAC TACACGCAGT ACCAACATGA CATTATGTGC ATCCGTAACT
	61 ACATCTTCCA CATACACCAA ATTCTGATTA TAAAGAGTAC ATGCGTCATG TGGAAGAATA
	121 TGATTTAAAG TTTATTTTTC
**GQ179958**	
	1 GAAAAATAAA CTTTAAATCA TATTCTTCCT CATGACGCAT GTACTCTTTA TAATCAGAAT
	61 TGGTGTATGT GGAAGATGTA GTTACGGATG CACATAATGT CATGTTGGTA CTGCGTGTAG
	121 TATCTACCAC AGTAACAAA
**GQ179959**	
	1 GAAAAATAAA CTTTAAATCA TATTCTTCCA TATGACGCAT GTACTCTTTA TAATCAGAAT
	61 TGGTGTATGT GGAAGATGTA GTTACGGATG CACATAATGT CATGTTGGTA CTGCGTGTAG
	121 TATCTACCAC AGTAACAAA

**Table 3.** Sequence Difference Among PCR products Detected

	1	2	3	4
**Sequence 1 (GQ179958)**	-	-	-	-
**Sequence 2 (GQ179959)**	2%	-	-	-
**Sequence 3 (GQ452049)**	1%	1%	-	-
**Sequence 4 (GQ452050)**	5%	5%	4%	-

## 5. Discussion

Worldwide, the second most common cancer in women is cervical cancer (3, 15). HPVs have been identified in 92.91% of a large series of cervical cancers collected internationally using PCR assays, of which 55 negative samples were tested again using HPV consensus GP5+/6+, E7 type-specific and CPI/II primers, where 72.7% were found to be positive for HPV. Thus, totally 97.2% of the cases were positive for HPV (14). In Norway, HPV was detected in 61% and 89% of neoplastic specimens from 1931 to 1960 and 1992 to 2004, respectively (3). 

Santos et al. (16) tested 198 cases with invasive carcinomas of cervix in Peru of which 173 (87.4%), 15 (7.6%) and 10 (5%) were diagnosed as squamous cell carcinoma, adenocarcinoma and adenosquamous cell carcinoma, respectively; HPV was detected in 94.9% of cases (95.3% in squamous cell carcinoma and 92% in adenocarcinoma/adenosquamous carcinomas). In this study only two cases were diagnosed as squamous cell carcinoma and no HPV was detected in these cases. This shows the low prevalence of this type of carcinoma in Isfahan, Iran. Due to the low number of cases, comparison with other data was not possible. Of 21 adenocarcinoma cases, five (23.8%) were positive for HPV. 

Forty percent (8) of the CIN cases (20) were positive for HPV. The rate of different carcinomas and also rate of HPV infection in each case were lower than other reports from different countries. This could be correlated to social behavior of women in the studied area, where women mostly have only one partner throughout their life, and also the rate of smoking behavior of women in the population studied. On the other hand the rate of HPV infection in chronic cervicitis cases was 78 out of 79 (98.7%), which is much higher than that reported by previous studies (17). As the same procedures were followed for all the tested cases, it is most unlikely that the positives scored in the cervicitis cases are due to cross contamination so the etiology remains to be established. 

To verify the presence of carcinoma cells in the samples used for PCR, different prepared paraffin-embedded biopsies were tested for histological evidence of tumors and along the blocks with carcinomas, blocks with normal tissues were also tested. In eight cases, the blocks with or without histopathological evidence for carcinoma were tested for the presence of HPV. HPV was detected in normal tissues while the carcinoma sections were negative. The failure to detect HPV DNA in these cervical carcinomas may have been due to integration of HPV DNA and disruption of PCR primer sequences or loss of HPV L1 ORF (18). On the other hand the hit and run mechanism which has already been explained for Bovine papillomavirus-4 may be involved in cervical cancers (19).
